# Supplementary material for: Secondary extended mannan side chains and attachment of the arabinan in mycobacterial lipoarabinomannan
Source: Commun Chem. 2020 Aug 7;3:101. doi: 10.1038/s42004-020-00356-3 (PMC8294699; doi:10.1038/s42004-020-00356-3)
Supplement: Supplementary file 2 — Descriptions of Additional Supplementary Files [file 42004_2020_356_MOESM2_ESM.pdf]

Description of Additional Supplementary Files

File Name: Supplementary Data 1
Description: Excel file showing area percent calculation of α-mannosidase-digested material of Msm-Ara-LM from M. smegmatis ΔMSMEG_6387 and deacylated Cellulomonas endo-arabinanase-digested LAM from M. tuberculosis.
