# Supplementary material for: Secondary extended mannan side chains and attachment of the arabinan in mycobacterial lipoarabinomannan
Source: Commun Chem. 2020 Aug 7;3:101. doi: 10.1038/s42004-020-00356-3 (PMC8294699; doi:10.1038/s42004-020-00356-3)
Supplement: Supplementary file 4 — Supplementary Information [file 42004_2020_356_MOESM4_ESM.pdf]

Secondary Extended Mannan Side Chains and Attachment of the Arabinan in Mycobacterial Lipoarabinomannan

Shiva K. Angala[1], Wei Li[1], Claudia M. Boot[2], Mary Jackson[1#*] and Michael R. McNeil[1#]

From the [1]Mycobacteria Research Laboratories, Department of Microbiology, Immunology and Pathology, Colorado State University, Fort Collins, CO 80523, USA, and the [2]Central Instrument Facility, Department of Chemistry, Colorado State University, Fort Collins, CO 80523, USA.

<u>Supplementary Note 1</u>: Preliminary control experiments to interpret the treatment of *M. smegmatis ΔMSMEG_6387* Ara-LM with α-mannosidase.

In the case of Msm-Ara-LM, we expected all internal mannosyl residues in the α-1,6 backbone between the T-Ara*f* residue and the inositol to not be susceptible to α-mannosidase. Thus, the enzyme removes the side chain α-mannosyl residues but not those in the backbone. To rightly interpret the data, we needed to determine the susceptibility of the α-Man*p* unit attached to *O*-2 of the inositol and thus a mixture of deacylated PIM_2 and PIM_6 were treated with α-mannosidase. LC/MS analysis revealed that PIM_1 was not produced, PIM_2 was essentially unchanged, and PIM_6 was converted to PIM_2.Thus the α-mannosidase does not remove mannosyl residues attached to inositol at either position 2 or 6. Therefore, the number of mannosyl residues in the α-1,6 mannan chain separating the Ara*f* from the inositol (whether or not they are substituted with an α-mannosyl residue at *O*-2) is equal to the number of mannosyl residues in the α-mannosidase treated LM minus one as the mannosyl residue on *O*-2 of the inositol residue is not removed. With this information, available, Msm-Ara-LM was treated with α-mannosidase. To confirm the lack of branched residues in the resulting products, MS/MS analysis was conducted on the triply charged $[M-(H)_3]^{3-}$ ion of non α-mannosidase digested *M. smegmatis* Ara_1-Man_{24}-Inos-P-Gro (*m/z* 1450.78) as a control, and the triply charged $[M-(H)_3]^{3-}$ ion of the α-mannosidase digested product Ara-Man_{17}-Inos-P-Gro (*m/z* 1072.66) (Supplementary Figure 7). A major contrast was noted between the two spectra, as in the α-mannosidase digested product Ara-Man_{17}-Inos-P-Gro the loss of a single mannosyl (presumably from *O*-2 of the inositol) to form the Y ion Ara_1Man_{16}-Inos-P-Gro was only very weakly observed but the loss of the non-reducing end Ara*f* residues to form the Y ion Man_{17}-Inos-P-Gro was abundantly formed (Supplementary Figure 7a). In contrast, the non-enzyme treated control, Ara_1Man_{24}-Inos-P-Gro (Supplementary Figure 7b) shows the opposite results where only very rarely is the Y ion Man_{24}-Inos-P-Gro is formed by loss of Ara observed but the Y ion Ara-Man_{23}-Inos-P-Gro formed by loss of a single mannosyl residue is abundant. Thus, the MS/MS data confirmed that the general structure of

the α-mannosidase products produced from Msm-Ara-LM were linear molecules with the arabinosyl residue at the non-reducing terminus.

Supplementary Data 1 (provided as a separate Excel file)

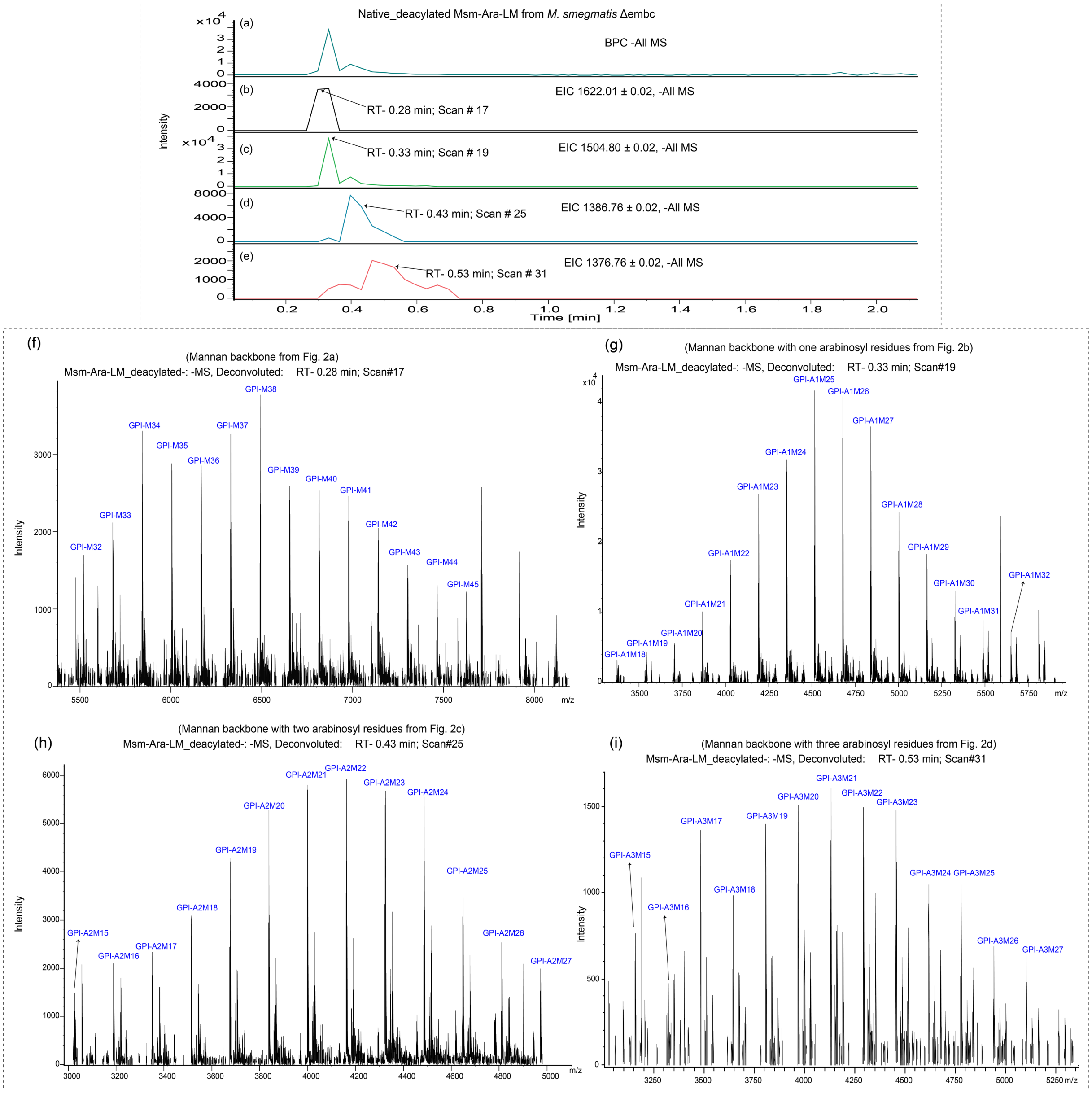

<u>Supplementary Figure 1</u>: LC/MS analysis of native deacylated Msm-Ara-LM from *M. smegmatis* Δ*embC*. Shown are base peak chromatograms (BPC) of Msm-Ara-LM in negative mode (a), extracted ion chromatograms (EICs) of GPI-Man_{38} at *m/z* 1622.01$[M\text{-}4H]^{4-}$ (b), GPI-Ara_1Man_{25} at *m/z* 1504.80 $[M\text{-}3H]^{3-}$ (c), GPI-Ara_2Man_{22} at *m/z* 1386.76 $[M\text{-}3H]^{3-}$ (d), and GPI-Ara_3Man_{21} at *m/z* 1376.76.80 $[M\text{-}3H]^{3-}$ (e). Multiply charged spectra from Fig. 2(a-d) are deconvoluted to singly charged $[M\text{-}H]^-$ spectra (f-i). The corresponding mass accuracy values for the individual compounds are shown in Supplementary Table 1.

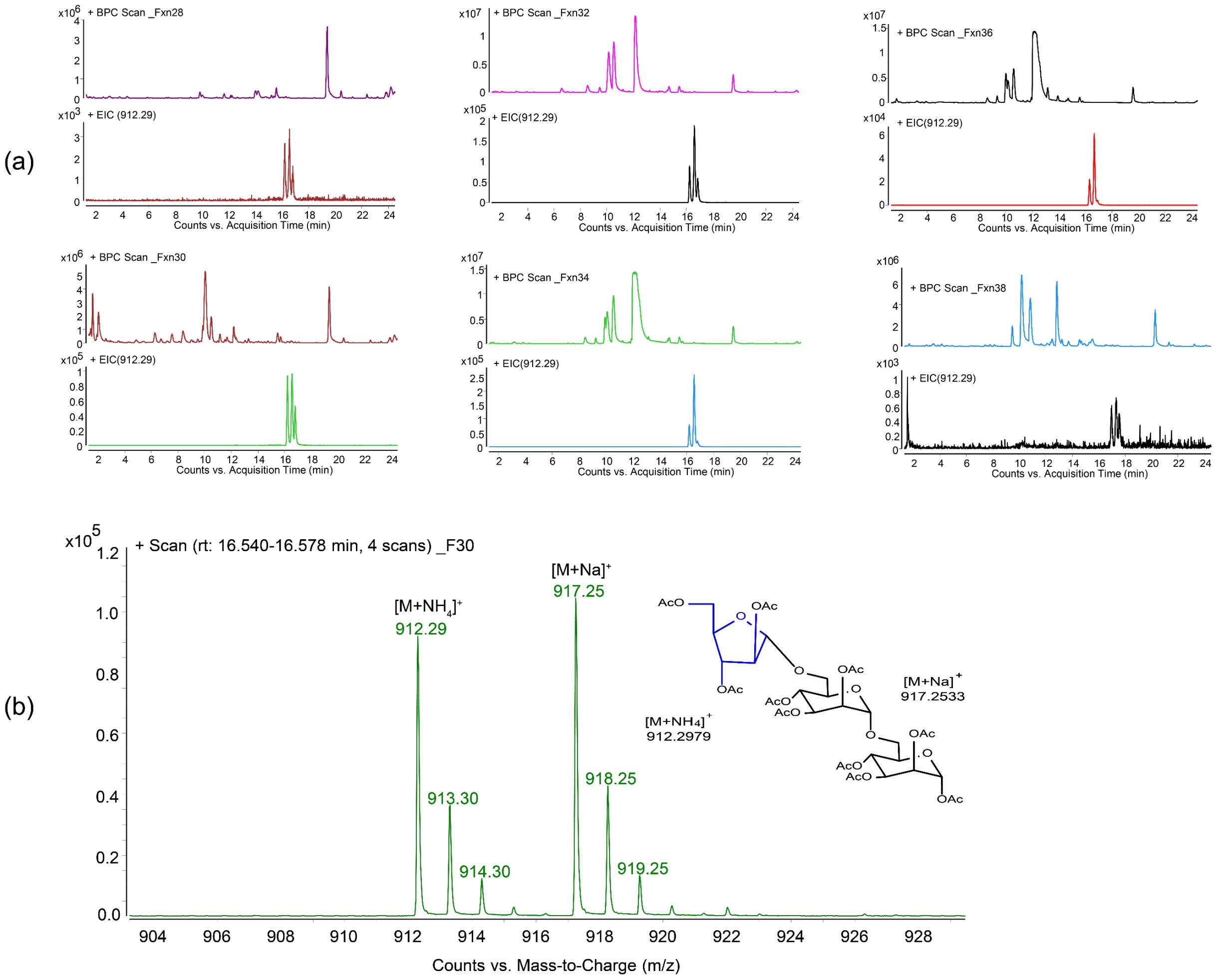

Supplementary Figure 2: LC/MS analysis of α-mannosidase and endomannanase-digested Msm-Ara-LM from *smegmatis* Δ*embC* in positive mode. (a) Shown are the BPCs and EICs of P2-column purified acetylated fractions. The extracted ion chromatogram (EIC) at *m/z* 912.29 shows the elution profile of acetylated products. (b) Mass spectrum of the peaks revealed acetylated Ara-Man-Man with the *m/z* values of 917.25 and 912.29 as sodiated and ammonium adducts.

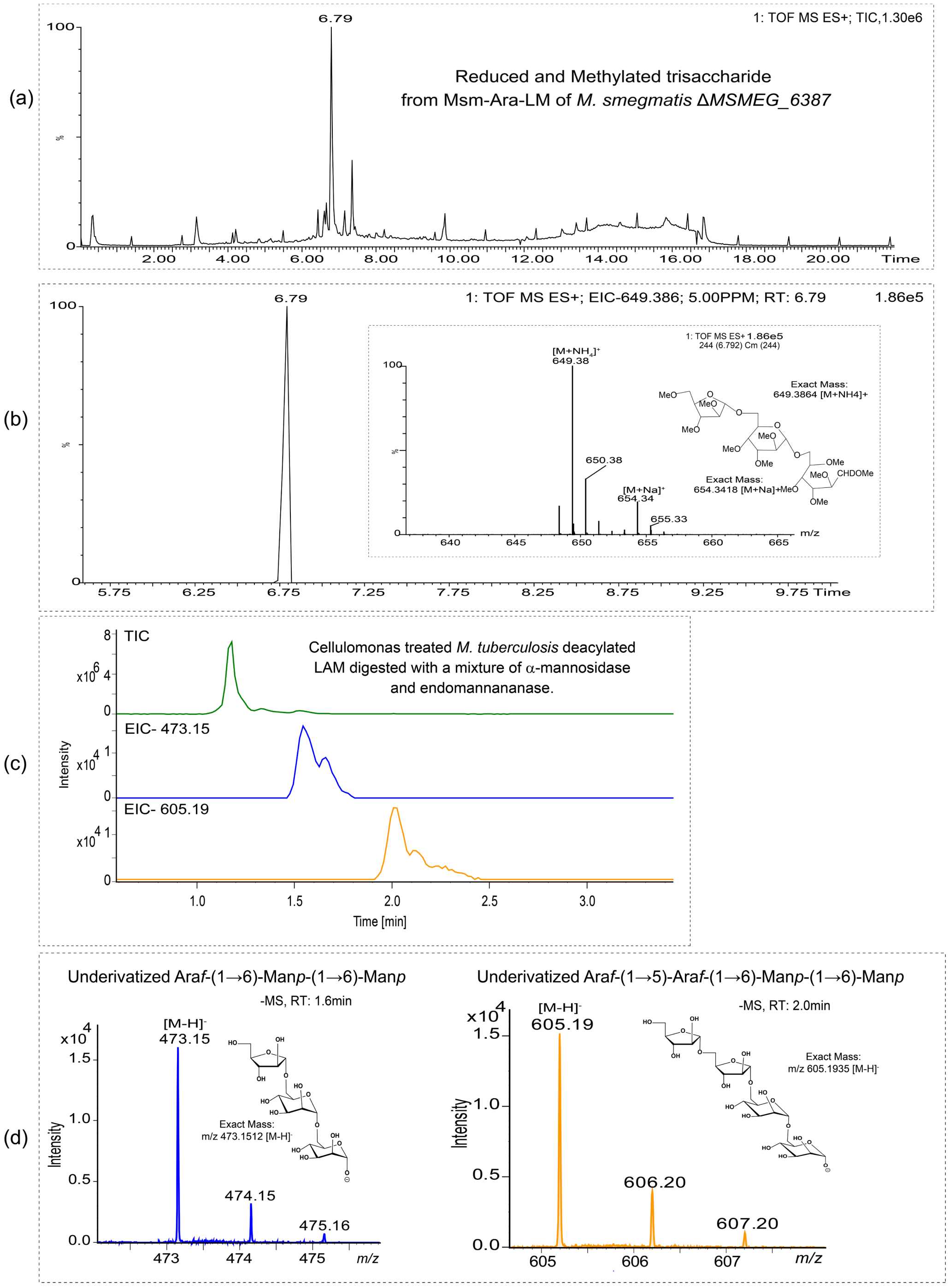

Supplementary Figure 3: (a) Shows the total ion chromatogram (TIC) of methylated Ara-Man-mannitol resulting from Msm-Ara-LM of *M. smegmatis ΔMSMEG_6387* in positive mode. (b) Extracted ion chromatogram at *m/z* 649.38, showing a single peak with a retention time (RT) 6.79 min representing Ara-Man-mannitol. Inset shows the structure and mass spectrum of reduced (deuterated), methylated Ara-

Man-mannitol with the *m/z* values of 654.34, and 649.38 as Na^+ and NH_4^+ adducts. Panel (c) shows TIC of *Cellulomonas* treated *M. tuberculosis* deacylated LAM after digestion with a mixture of α-mannosidase and endo 1,6-α-mannanase obtained in negative mode. The EICs of *m/z* 473.15 and 605.19 shown in panel (c) correspond to the non-derivatized trisaccharide and tetrasaccharide. Their mass spectra of $[M-H]^-$ ions are shown in panel (d).

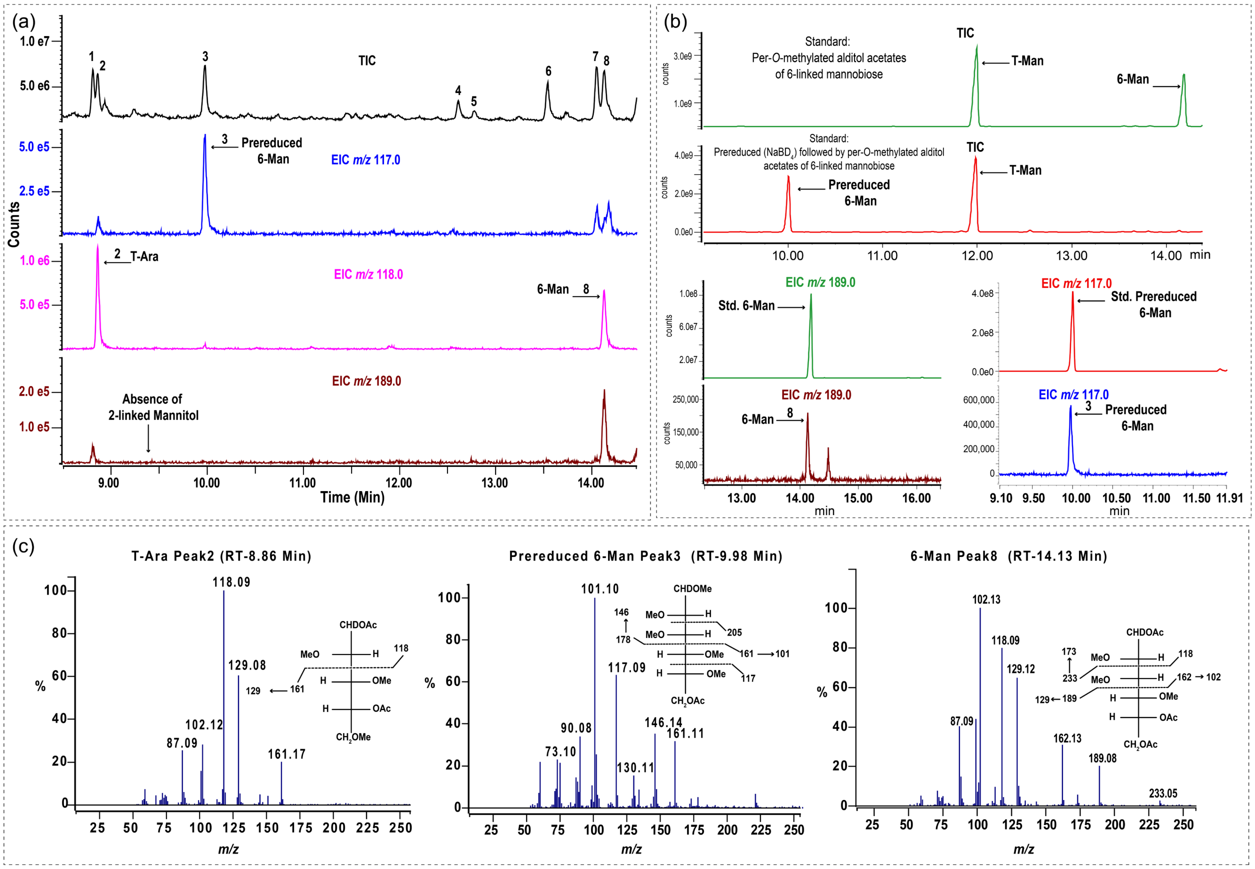

Supplementary Figure 4: GC/MS analysis of the partially *O*-methylated partially *O*-acetylated alditols derived from the Ara_1Man_2 trisaccharide isolated from enzymatic degradation of Msm-Ara-LM produced by *M. smegmatis ΔMSMEG_6387*. (a) Peaks 1, 4, 5, 6, and 7 labeled on the TIC are non-carbohydrate contaminants (due to the low amounts of trisaccharide analyzed), and peaks 2, 3, and 8 are the partially-*O*-methylated partially-*O*-acetylated alditols. (b) The two hexitols were shown to be in the *manno*-configuration by comparison with standards. (c) The MS spectra of the 1,4-di-*O*-acetyl 2,3,5-tri-*O*-methyl arabinitol, 6-*O*-acetyl 1,2,3,4,5 penta-*O*-methyl mannitol and 1,5,6-tri-*O*-acetyl 2,3,4-tri-*O*-methyl mannitol.

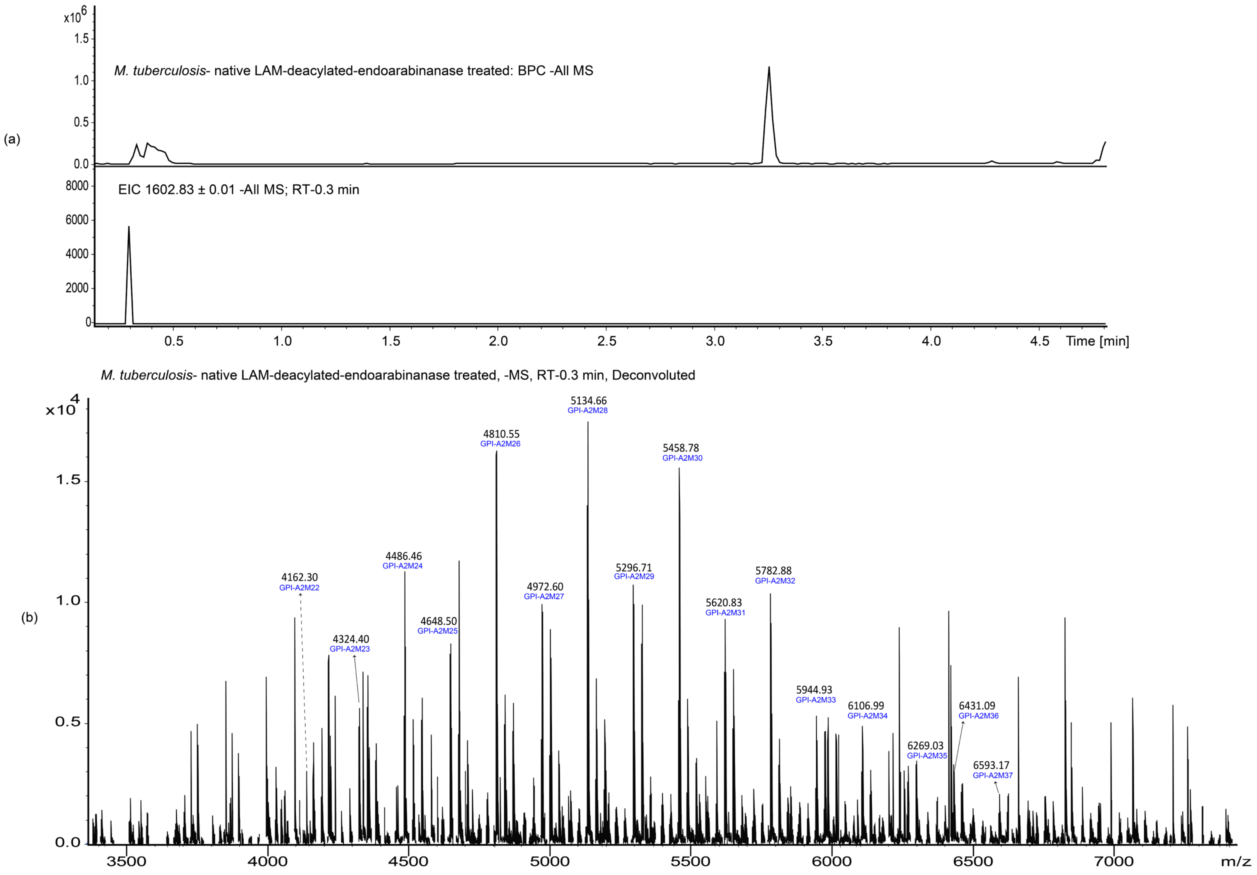

<u>Supplementary Figure 5:</u> LC/MS analysis of *Cellulomonas* endo-arabinanase digested native*,* deacylated *M. tuberculosis* LAM. (a) Shown are the BPC, and EIC obtained in the negative mode for the triply charged dominant ion at *m/z* 1602.83 $[M-3H]^{3-}$. The EIC generated represents the GPI-Ara_2Man_{26} show in Fig. 4. (b) Deconvoluted mass spectrum obtained at 0.3 min retention time show dominant signals for Ara_2Manx-Gro in the digestion mixture. Mass accuracy (ppm) values for the corresponding deconvoluted spectrum are shown in Supplementary Table 3.

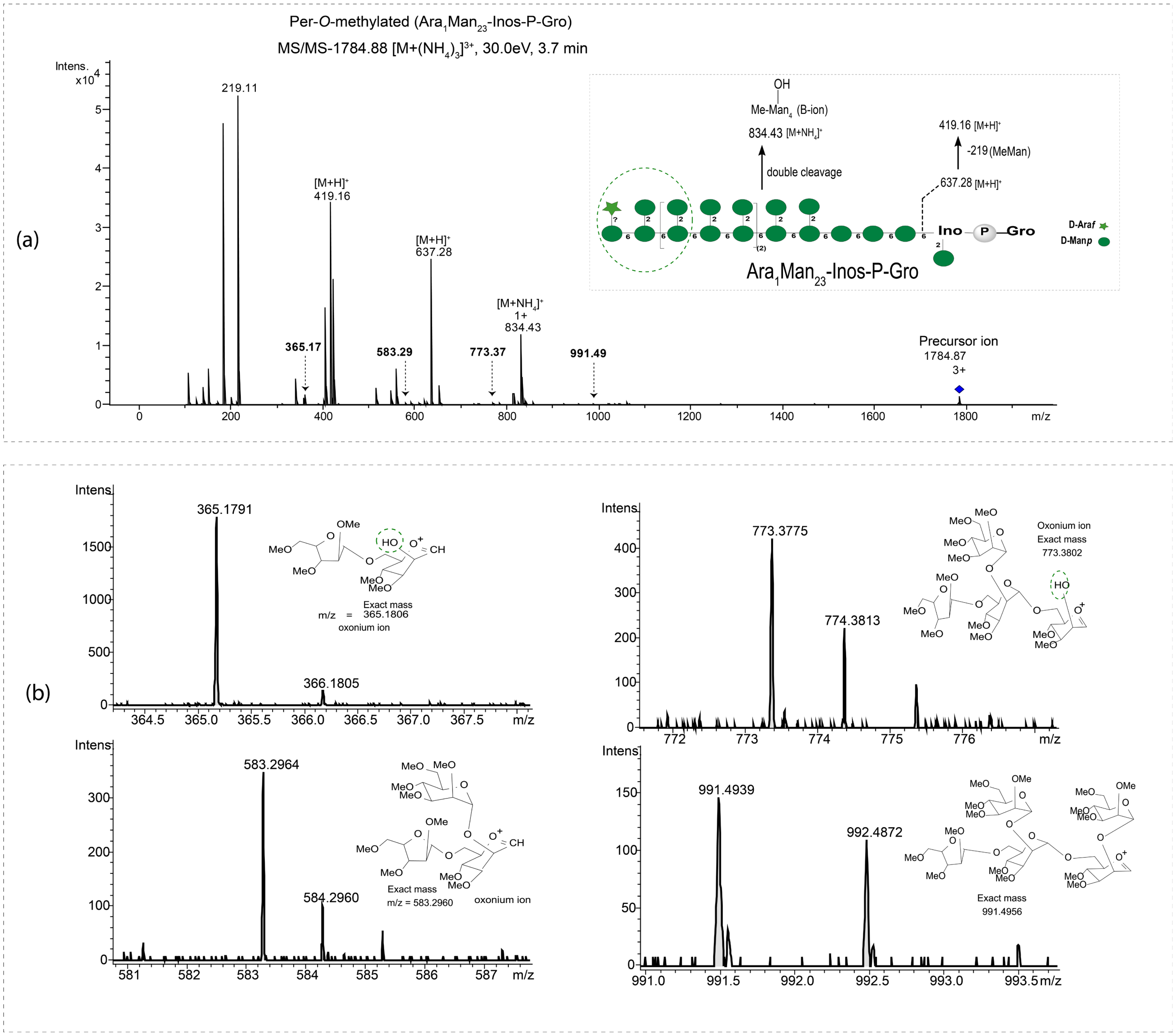

Supplementary Figure 6: (Panel a) LC-MS/MS spectrum of triply charged $(NH_4+)^{3+}$ per-*O*-methylated Ara_1-Man_{23}-Inos-P-Gro at *m/z* 1784.89. The interpretation of observed singly charged fragment ions (most abundant) is presented in the inset structure. The vertical dotted arrows mark the low mass oxonium ions. Panel (b) shows a mass spectrum for all low mass oxonium ions identified, demonstrating the clear fragmentation pattern of the molecule at the site of arabinose attachment on the mannan backbone. Assignments of fragment oxonium ions are illustrated as insets (b).

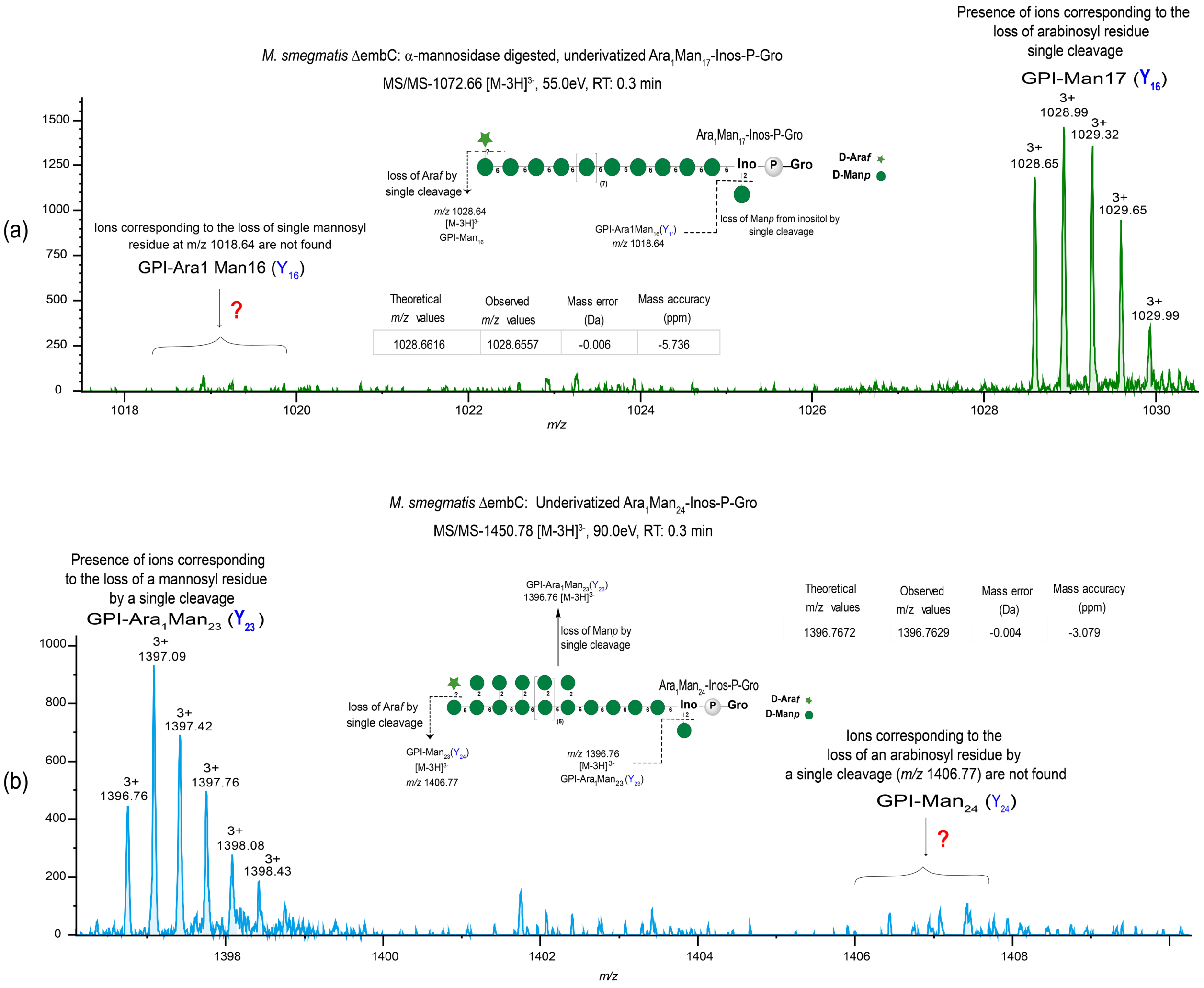

<u>Supplementary Figure 7</u>: A comparison of the loss of mannosyl versus arabinosyl residues in the MS/MS spectra on the triple charged $[M-(H)_3]^{3-}$ ions (a) at *m/z* 1072.66 of underivatized Ara$_1$Man$_{18}$-Inos-P-Gro produced by α-mannosidase treatment of Msm-Ara-LM from *M. smegmatis ΔMSMEG_6387* and (b) at *m/z* 1450.78 of underivatized Ara$_1$Man$_{24}$ Inos-P-Gro from *M. smegmatis ΔMSMEG_6387* that was not treated with α-mannosidase. (See Supplemental data 1 for more details). Interpretation of observed fragments ions by single cleavage are illustrated as insets.

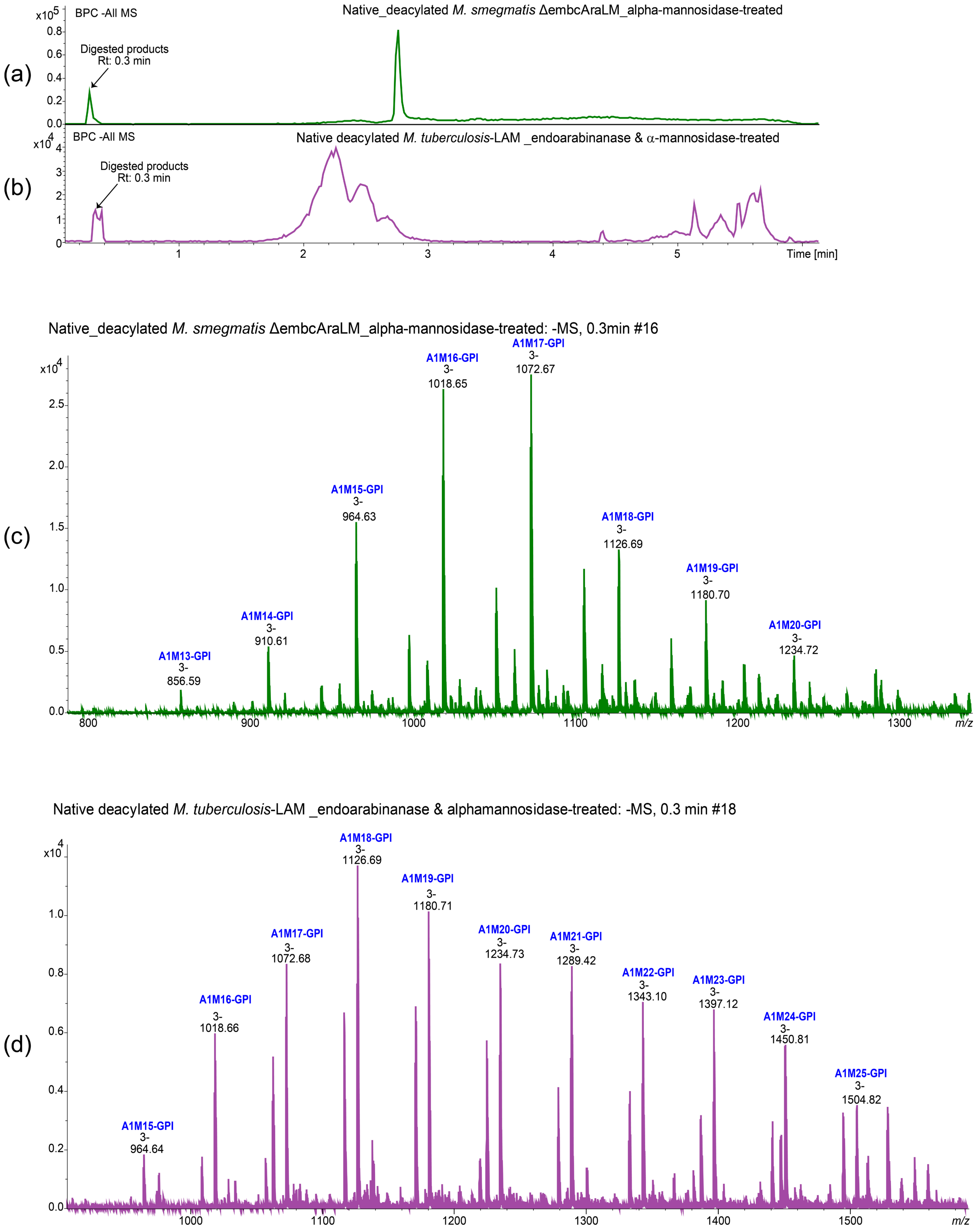

<u>**Supplementary Figure 8**</u>**:** LC/MS analysis showing base peak chromatograms of the α-mannosidase digestion products from (a) Msm-Ara-LM, and (b) *Cellulomonas* endo-arabinanase-digested *M. tuberculosis* LAM. Their corresponding mass spectra are obtained in negative mode (c-d). Mass spectrum

from (c) Msm-Ara-LM shows the presence of triply charged ions $[M-3H]^{3-}$ for 13-18 mannosyl residues between the non-reducing end of arabinose and inositol. Mass spectrum from (d) *Cellulomonas* endo-arabinanase digested *M. tuberculosis* LAM shows a series of triply charged ions $[M-3H]^{3-}$ for 15-25 mannosyl residues between the tenon-reducing end of terminal arabinose and inositol.

(a)

(b)

(c)

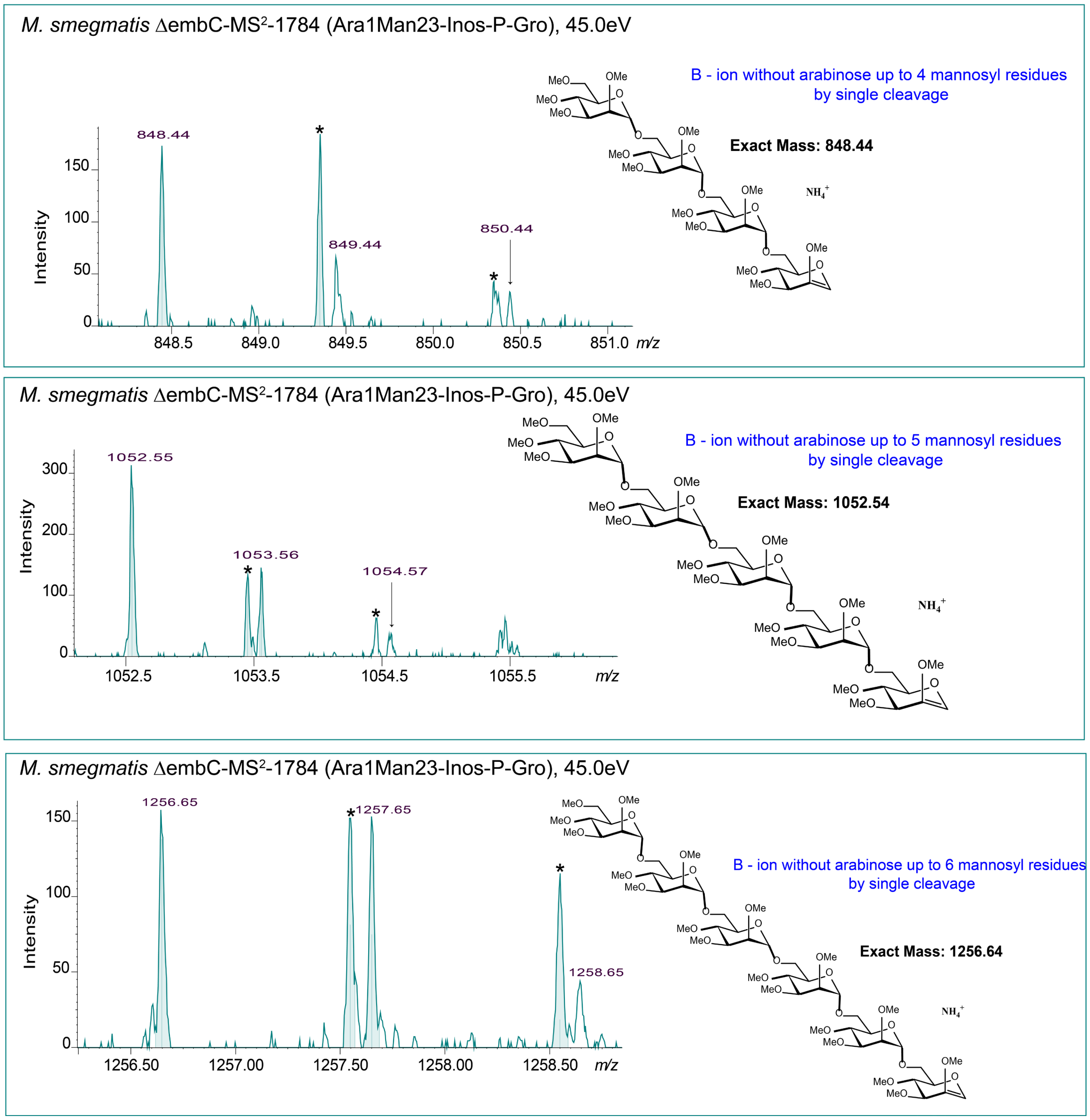

<u>Supplementary Figure 9</u>: A low mass portion of the MS/MS spectrum of the triple charged $[M+(NH_4)_3]^{3+}$ ion at *m/z* 1784.8 produced by the per-*O*-methylated Ara_1Man_{23}-Inos-P-Gro molecule by *M. smegmatis ΔMSMEG_6387* showing diagnostic B ions and the rationale for their formation. (a) *m/z* 848.44 made up of 4 mannosyl residues, (b) *m/z* 1052.56 made up of 5 mannosyl residues, and (c) *m/z* 1256.64 made up of 6 mannosyl residues. These mannosyl residues are shown in a linear arrangement for clarity, but the actual arrangement of the mannosyl residues (*i.e.,* branching pattern) is not yet known. These B ions, although of low intensity, confirm the extended mannan backbone side chains. The peaks with an asterisk are from other unidentified ions.

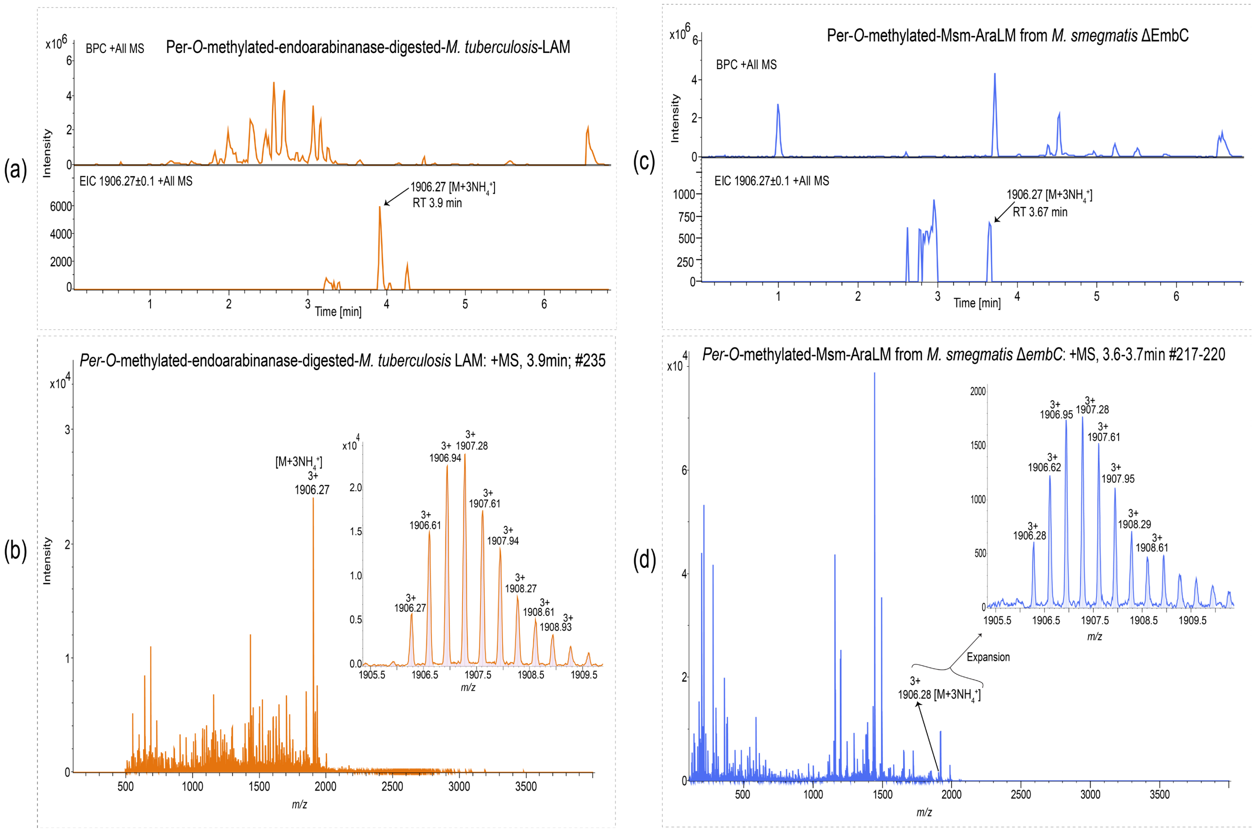

Supplementary Figure 10: BPCs and EICs (*m/z* 1906.27) of the per-*O*-methylated Ara_2Man_{24}-Inos-P-Gro molecule produced by *Cellulomonas*-digested *M. tuberculosis*-LAM (a) and *M. smegmatis ΔMSMEG_6387* (c) were obtained in positive mode. Panels b and d shows their corresponding MS spectra of the triple charged $[M+(NH_4)_3]^{3+}$ ion at *m/z* value of 1906.27.

	Glyerol phosphate-Inositol (GPI)-with varying number of mannyosyl/arabinosyl residues on the mannan backbone		Charge	Theoretical mass (*m/z*)	Observed mass (*m/z*)	Mass error (Da)	Mass accuracy (ppm)
Mannan backbone [a]	GPI-M33	GPI-Man33	$[M-4H^+]^{4-}$	1419.4451	1419.4468	0.002	1.2
	GPI-M34	GPI-Man34	$[M-4H^+]^{4-}$	1459.9583	1459.9641	0.006	4.0
	GPI-M35	GPI-Man35	$[M-4H^+]^{4-}$	1500.4715	1500.4797	0.008	5.4
	GPI-M36	GPI-Man36	$[M-4H^+]^{4-}$	1540.9847	1540.9903	0.006	3.6
	GPI-M37	GPI-Man37	$[M-4H^+]^{4-}$	1581.4979	1581.5055	0.008	4.8
	GPI-M38	GPI-Man38	$[M-4H^+]^{4-}$	1622.0111	1622.0212	0.010	6.2
	GPI-M39	GPI-Man39	$[M-4H^+]^{4-}$	1662.5243	1662.5288	0.004	2.7
	GPI-M40	GPI-Man40	$[M-4H^+]^{4-}$	1703.0375	1703.0424	0.005	2.8
	GPI-M41	GPI-Man41	$[M-4H^+]^{4-}$	1743.5508	1743.5567	0.006	3.4
	GPI-M42	GPI-Man42	$[M-4H^+]^{4-}$	1784.0640	1784.0660	0.002	1.1
	GPI-M43	GPI-Man43	$[M-4H^+]^{4-}$	1824.5772	1824.5951	0.018	9.8
	GPI-M44	GPI-Man44	$[M-4H^+]^{4-}$	1865.0904	1865.1008	0.010	5.6
	GPI-M45	GPI-Man45	$[M-4H^+]^{4-}$	1905.6036	1905.6057	0.002	1.1
	GPI-M46	GPI-Man46	$[M-4H^+]^{4-}$	1946.1168	1946.1314	0.015	7.5
	GPI-M47	GPI-Man47	$[M-4H^+]^{4-}$	1986.6300	1986.6356	0.006	2.8
	GPI-M48	GPI-Man48	$[M-4H^+]^{4-}$	2027.1432	2027.1602	0.017	8.4
Mannan backbone with one arabinose [b]	GPI-M2	GPI-Man2	$[M-H^+]^{-}$	657.1649	657.1670	0.002	3.2
	GPI-M3	GPI-Man3	$[M-H^+]^{-}$	819.2177	819.2218	0.004	5.0
	GPI-M4	GPI-Man4	$[M-H^+]^{-}$	981.2705	981.2735	0.003	3.0
	GPI-M5	GPI-Man5	$[M-H^+]^{-}$	1143.3233	1143.3276	0.004	3.7
	GPI-A1M17	GPI-Ara1Man17	$[M-3H^+]^{3-}$	1072.6616	1072.6688	0.007	6.7
	GPI-A1M18	GPI-Ara1Man18	$[M-3H^+]^{3-}$	1126.6792	1126.6830	0.004	3.3
	GPI-A1M19	GPI-Ara1Man19	$[M-3H^+]^{3-}$	1180.6968	1180.6993	0.002	2.1
	GPI-A1M20	GPI-Ara1Man20	$[M-3H^+]^{3-}$	1234.7144	1234.7209	0.006	5.2
	GPI-A1M21	GPI-Ara1Man21	$[M-3H^+]^{3-}$	1288.7321	1288.7385	0.006	5.0
	GPI-A1M22	GPI-Ara1Man22	$[M-3H^+]^{3-}$	1342.7497	1342.7559	0.006	4.6
	GPI-A1M23	GPI-Ara1Man23	$[M-3H^+]^{3-}$	1396.7673	1396.7740	0.007	4.8
	GPI-A1M24	GPI-Ara1Man24	$[M-3H^+]^{3-}$	1450.7849	1450.7917	0.007	4.7
	GPI-A1M25	GPI-Ara1Man25	$[M-3H^+]^{3-}$	1504.8025	1504.8094	0.007	4.6
	GPI-A1M26	GPI-Ara1Man26	$[M-3H^+]^{3-}$	1558.8201	1558.8270	0.007	4.4
	GPI-A1M27	GPI-Ara1Man27	$[M-3H^+]^{3-}$	1612.8377	1612.8451	0.007	4.6
	GPI-A1M28	GPI-Ara1Man28	$[M-3H^+]^{3-}$	1666.8553	1666.8618	0.007	3.9
	GPI-A1M29	GPI-Ara1Man29	$[M-3H^+]^{3-}$	1720.8729	1720.8788	0.006	3.4
	GPI-A1M30	GPI-Ara1Man30	$[M-3H^+]^{3-}$	1774.8905	1774.8992	0.009	4.9
	GPI-A1M31	GPI-Ara1Man31	$[M-3H^+]^{3-}$	1828.9081	1828.9165	0.008	4.6
	GPI-A1M32	GPI-Ara1Man32	$[M-3H^+]^{3-}$	1882.9257	1882.9334	0.008	4.1
	GPI-A1M33	GPI-Ara1Man33	$[M-3H^+]^{3-}$	1936.9433	1936.9514	0.008	4.2
	GPI-A1M34	GPI-Ara1Man34	$[M-3H^+]^{3-}$	1990.9609	1990.9727	0.012	5.9
Mannan backbone with two arabinoses [c]	GPI-A2M15	GPI-Ara2Man15	$[M-3H^+]^{3-}$	1008.6405	1008.6443	0.004	3.8
	GPI-A2M16	GPI-Ara2Man16	$[M-3H^+]^{3-}$	1062.6581	1062.6648	0.007	6.3
	GPI-A2M17	GPI-Ara2Man17	$[M-3H^+]^{3-}$	1116.6757	1116.6807	0.005	4.5
	GPI-A2M18	GPI-Ara2Man18	$[M-3H^+]^{3-}$	1170.6933	1170.6995	0.006	5.3
	GPI-A2M19	GPI-Ara2Man19	$[M-3H^+]^{3-}$	1224.7109	1224.7169	0.006	4.9
	GPI-A2M20	GPI-Ara2Man20	$[M-3H^+]^{3-}$	1278.7285	1278.7347	0.006	4.8
	GPI-A2M21	GPI-Ara2Man21	$[M-3H^+]^{3-}$	1332.7461	1332.7531	0.007	5.2
	GPI-A2M22	GPI-Ara2Man22	$[M-3H^+]^{3-}$	1386.7637	1386.7719	0.008	5.9
	GPI-A2M23	GPI-Ara2Man23	$[M-3H^+]^{3-}$	1440.7814	1440.7858	0.004	3.1
	GPI-A2M24	GPI-Ara2Man24	$[M-3H^+]^{3-}$	1494.7990	1494.8064	0.007	5.0
	GPI-A2M25	GPI-Ara2Man25	$[M-3H^+]^{3-}$	1548.8166	1548.8256	0.009	5.8
	GPI-A2M26	GPI-Ara2Man26	$[M-3H^+]^{3-}$	1602.8342	1602.8408	0.007	4.1
	GPI-A2M27	GPI-Ara2Man27	$[M-3H^+]^{3-}$	1656.8518	1656.8618	0.010	6.0
	GPI-A2M28	GPI-Ara2Man28	$[M-3H^+]^{3-}$	1710.8694	1710.8849	0.016	9.1
	GPI-A2M29	GPI-Ara2Man29	$[M-3H^+]^{3-}$	1764.8870	1764.8912	0.004	2.4
	GPI-A2M30	GPI-Ara2Man30	$[M-3H^+]^{3-}$	1818.9046	1818.9047	0.000	0.1
	GPI-A2M31	GPI-Ara2Man31	$[M-3H^+]^{3-}$	1872.9222	1872.9249	0.003	1.4
Mannan backbone with three arabinoses [d]	GPI-A3M15	GPI-Ara3Man15	$[M-3H^+]^{3-}$	1052.6546	1052.6597	0.005	4.9
	GPI-A3M16	GPI-Ara3Man16	$[M-3H^+]^{3-}$	1106.6722	1106.6767	0.005	4.1
	GPI-A3M17	GPI-Ara3Man17	$[M-3H^+]^{3-}$	1160.6898	1160.6965	0.007	5.8
	GPI-A3M18	GPI-Ara3Man18	$[M-3H^+]^{3-}$	1214.7074	1214.7139	0.006	5.3
	GPI-A3M19	GPI-Ara3Man19	$[M-3H^+]^{3-}$	1268.7250	1268.7357	0.011	8.4
	GPI-A3M20	GPI-Ara3Man20	$[M-3H^+]^{3-}$	1322.7426	1322.7491	0.006	4.9
	GPI-A3M21	GPI-Ara3Man21	$[M-3H^+]^{3-}$	1376.7602	1376.7651	0.005	3.5
	GPI-A3M22	GPI-Ara3Man22	$[M-3H^+]^{3-}$	1430.7778	1430.7860	0.008	5.7
	GPI-A3M23	GPI-Ara3Man23	$[M-3H^+]^{3-}$	1484.7954	1484.8052	0.010	6.6
	GPI-A3M24	GPI-Ara3Man24	$[M-3H^+]^{3-}$	1538.8130	1538.8173	0.004	2.8
	GPI-A3M25	GPI-Ara3Man25	$[M-3H^+]^{3-}$	1592.8307	1592.8371	0.006	4.0
	GPI-A3M26	GPI-Ara3Man26	$[M-3H^+]^{3-}$	1646.8483	1646.8562	0.008	4.8
	GPI-A3M27	GPI-Ara3Man27	$[M-3H^+]^{3-}$	1700.8659	1700.8700	0.004	2.4
	GPI-A3M28	GPI-Ara3Man28	$[M-3H^+]^{3-}$	1754.8835	1754.8944	0.011	6.2

[a] The ions identified in Fig. 2a; [b] The ions identified in Fig. 2b; [c] The ions identified in Fig. 2c [d] The ions identified in Fig. 2d

Supplementary Table 1. Assignment of major ionic species found in the complex mixture of Msm-Ara-LM from *M. smegmatis ΔMSMEG_6387* (see Fig 2a-d)**.**

	MS/MS Fragment ions	Charge	Theoretical mass (*m/z*)	Observed mass (*m/z*)	Mass error (Da)	Mass accuracy (ppm)
Ara1-Man2-Reduced-Methylated[a]	B_1	$[M+Na]^+$	197.0784	197.0769	-0.001	-7.61
	B_2	$[M+Na]^+$	401.1782	401.1681	-0.010	-25.18
	C_1	$[M+Na]^+$	215.0890	215.0903	0.001	6.04
	C_2	$[M+Na]^+$	419.1888	419.1884	0.000	-0.95
	Y_1	$[M+Na]^+$	276.1528	276.1535	0.001	2.53
	Y_2	$[M+Na]^+$	480.2526	480.2548	0.002	4.58
	$^{0,4}A_2$	$[M+Na]^+$	257.0996	257.0650	-0.035	-134.58
	$^{3,5}A_2$	$[M+Na]^+$	285.1309	285.1376	0.007	23.50
	$^{3,5}A_3$	$[M+Na]^+$	489.2306	489.2347	0.004	8.38
Ara1-Man2 underivatized[b]	B_1	$[M-H]^-$	131.0350	131.0356	-0.001	-4.58
	C_1	$[M-H]^-$	149.0455	149.0467	-0.001	-8.05
	C_2	$[M-H]^-$	311.0984	311.1003	-0.002	-6.11
	$^{0,4}A_2$	$[M-H]^-$	191.0561	191.0573	-0.001	-6.28
	$^{0,3}A_2$	$[M-H]^-$	221.0667	221.0681	-0.001	-6.33
	$^{0,2}A_2$	$[M-H]^-$	251.0772	251.0789	-0.002	-6.77
	$^{0,4}A_3$	$[M-H]^-$	353.1089	353.1109	-0.002	-5.66
	$^{0,2}A_3$	$[M-H]^-$	413.1301	413.1312	-0.001	-2.66
Ara2-Man2 underivatized[c]	C_1	$[M-H]^-$	149.0455	149.0453	0.000	1.34
	C_2	$[M-H]^-$	281.0878	281.0886	-0.001	-2.85
	C_3	$[M-H]^-$	443.1406	443.1415	-0.001	-2.03
	$^{0,3}A_2$	$[M-H]^-$	191.0561	191.0569	-0.001	-4.19
	$^{0,4}A_3$	$[M-H]^-$	323.0984	323.0984	0.000	0.00
	$^{0,3}A_3$	$[M-H]^-$	353.1089	353.1085	0.000	1.13
	$^{0,2}A_3$	$[M-H]^-$	383.1195	383.1199	0.000	-1.04
	$^{0,4}A_4$	$[M-H]^-$	485.1512	485.1513	0.000	-0.21
	$^{0,2}A_4$	$[M-H]^-$	545.1723	545.1722	0.000	0.18

a The ions identified in Fig. 3a   b The ions identified in Fig. 3b   c The ions identified in Fig. 3c

<u>Supplementary Table 2</u>: Structural assignment of MS/MS fragment ions for the reduced-methylated Ara-Man-mannitol (positive mode), native underivatized tri-Ara-Man-Man and tetra Ara-Ara-Man-Man (negative mode) from Fig. 3(a-c).

	Glyerol phosphate-Inositol (GPI)-with varying number of mannosyl residues in the mannan backbone		Theoretical mass (*m/z*) $[M-H]^-$	Observed mass (*m/z*) $[M-H]^-$	Mass error (Da)	Mass accuracy (ppm)
Mannan backbone [a]	GPI-M23	GPI-Man23	4060.2741	4060.3060	0.032	7.86
	GPI-M24	GPI-Man24	4222.3269	4222.3767	0.050	11.79
	GPI-M25*	GPI-Man25	4384.3797	4384.3797	0.000	0.00
	GPI-M26	GPI-Man26	4546.4326	4546.4730	0.040	8.90
	GPI-M27	GPI-Man27	4708.4854	4708.5270	0.042	8.84
	GPI-M28	GPI-Man28	4870.5382	4870.5924	0.054	11.13
	GPI-M29	GPI-Man29	5032.5910	5032.6049	0.014	2.76
	GPI-M30	GPI-Man30	5194.6438	5194.6990	0.055	10.62
	GPI-M31*	GPI-Man31	5356.6967	5356.7445	0.048	8.93
	GPI-M32	GPI-Man32	5518.7495	5518.8071	0.058	10.44
	GPI-M33	GPI-Man33	5680.8023	5680.8634	0.061	10.76
	GPI-M34	GPI-Man34	5842.8551	5842.9198	0.065	11.07
	GPI-M35	GPI-Man35	6004.9079	6004.9685	0.061	10.09
	GPI-M36	GPI-Man36	6166.9608	6166.9973	0.037	5.93
	GPI-M37[nd]	GPI-Man37[nd]	6329.0136	_	__	_
	GPI-M38	GPI-Man38	6491.0664	6491.1116	0.045	6.96
Mannan backbone with one arabinose [b]	GPI-A1M20	GPI-Ara1Man20	3706.15789	3706.2075	0.050	13.39
	GPI-A1M21	GPI-Ara1Man21	3868.21071	3868.2546	0.044	11.35
	GPI-A1M22	GPI-Ara1Man22	4030.26353	4030.3085	0.045	11.16
	GPI-A1M23	GPI-Ara1Man23	4192.31635	4192.3663	0.050	11.91
	GPI-A1M24	GPI-Ara1Man24	4354.36917	4354.4082	0.039	8.96
	GPI-A1M25	GPI-Ara1Man25	4516.42199	4516.4617	0.040	8.79
	GPI-A1M26	GPI-Ara1Man26	4678.47481	4678.5243	0.049	10.58
	GPI-A1M27	GPI-Ara1Man27	4840.52763	4840.5771	0.049	10.22
	GPI-A1M28	GPI-Ara1Man28	5002.58045	5002.6271	0.047	9.33
	GPI-A1M29	GPI-Ara1Man29	5164.63327	5164.6787	0.045	8.80
	GPI-A1M30	GPI-Ara1Man30	5326.68609	5326.7313	0.045	8.49
	GPI-A1M31	GPI-Ara1Man31	5488.73891	5488.7978	0.059	10.73
	GPI-A1M32	GPI-Ara1Man32	5650.79173	5650.8497	0.058	10.26
	GPI-A1M33	GPI-Ara1Man33	5812.84455	5812.9088	0.064	11.05
	GPI-A1M34	GPI-Ara1Man34	5974.89737	5974.9261	0.029	4.81
	GPI-A1M35	GPI-Ara1Man35	6136.95019	6137.025	0.075	12.19
	GPI-A1M36	GPI-Ara1Man36	6299.00301	6299.0444	0.041	6.57
	GPI-A1M37*	GPI-Ara1Man37*	6461.05583	_	_	-
	GPI-A1M38	GPI-Ara1Man38	6623.10865	6623.1428	0.034	5.16
	GPI-A1M39	GPI-Ara1Man39	6785.16147	6785.1998	0.038	5.65
	GPI-A1M40*	GPI-Ara1Man40*	6947.21429	_	_	_
	GPI-A1M41	GPI-Ara1Man41	7109.26711	7109.3309	0.064	8.97
Mannan backbone with two arabinose [c]	GPI-A2M17	GPI-Ara2Man17	3352.04169	3352.0776	0.036	10.71
	GPI-A2M18	GPI-Ara2Man18	3514.09451	3514.128	0.033	9.53
	GPI-A2M19	GPI-Ara2Man19	3676.14733	3676.2009	0.054	14.57
	GPI-A2M20*	GPI-Ara2Man20*	3838.20015	_	_	_
	GPI-A2M21*	GPI-Ara2Man21*	4000.25297	_	_	_
	GPI-A2M22	GPI-Ara2Man22	4162.30579	4162.3511	0.045	10.89
	GPI-A2M23	GPI-Ara2Man23	4324.35861	4324.4047	0.046	10.66
	GPI-A2M24	GPI-Ara2Man24	4486.41143	4486.4635	0.052	11.61
	GPI-A2M25	GPI-Ara2Man25	4648.46425	4648.5049	0.041	8.74
	GPI-A2M26	GPI-Ara2Man26	4810.51707	4810.5547	0.038	7.82
	GPI-A2M27	GPI-Ara2Man27	4972.56989	4972.6001	0.030	6.08
	GPI-A2M28	GPI-Ara2Man28	5134.62271	5134.6659	0.043	8.41
	GPI-A2M29	GPI-Ara2Man29	5296.67553	5296.7111	0.036	6.72
	GPI-A2M30	GPI-Ara2Man30	5458.72835	5458.7807	0.052	9.59
	GPI-A2M31	GPI-Ara2Man31	5620.78117	5620.8397	0.059	10.41
	GPI-A2M32	GPI-Ara2Man32	5782.83399	5782.8893	0.055	9.56
	GPI-A2M33	GPI-Ara2Man33	5944.88681	5944.9302	0.043	7.30
	GPI-A2M34	GPI-Ara2Man34	6106.93963	6106.999	0.059	9.72
	GPI-A2M35	GPI-Ara2Man35	6268.99245	6269.0302	0.038	6.02
	GPI-A2M36	GPI-Ara2Man36	6431.04527	6431.0945	0.049	7.66
	GPI-A2M37	GPI-Ara2Man37	6593.09809	6593.1743	0.076	11.56
	GPI-A2M38*	GPI-Ara2Man38*	6755.15091	_	_	_
	* merged	nd-not detected				

a, b, & c The ions obtained after deconvolution of mass spectrum from Fig. 4

<u>Supplementary Table 3</u>. Assignment of major ionic species found in the complex mixture of *Cellulomonas* endo-arabinanase digested native, deacylated *M. tuberculosis* LAM.

Per-O-methylated (Ara2Man24-Inos-P-Gro) [a]
MS/MS-1906.27 $[M+(NH_4)_3]^{3+}$

Varying number of Mannosyl residues	Single Cleavage Y-ions	Charge Status (NH4+ adduct)	Theoretical m/z values	*M. tuberculosis* Observed m/z values	*M. tuberculosis* mass accuracy (ppm)	*M. smegmatis ΔembC* Observed m/z values	*M. smegmatis ΔembC* mass accuracy (ppm)
Ara2Man20	Y20-$(OH)_1$	2	2435.1931	2435.6884	203.4	2435.1858	-3.0
Ara2Man21	Y21-$(OH)_1$	2	2537.243	2537.2352	-3.1	2537.2458	1.1
Ara2Man22	Y22-$(OH)_1$	2	2639.2929	2639.2932	0.1	2639.286	-2.6
Ara2Man23	Y23-$(OH)_1$	2	2741.3428	2741.3298	-4.7	2741.3553	4.6
Ara2Man19	Y19-$(OH)_2$	2	2326.1354	2326.6331	214.0	2326.1175	-7.7
Ara2Man20	Y20-$(OH)_2$	2	2428.1853	2428.1723	-5.3	2428.1818	-1.4
Ara2Man21	Y21-$(OH)_2$	2	2530.2351	2530.2395	1.7	2530.2378	1.1
Ara2Man22	Y22-$(OH)_2$	2	2632.285	2632.2897	1.8	2632.2762	-3.4
Ara1Man20	Y20-$(OH)_1$	2	2355.1563	nd	_	2355.1635	3.0
Ara1Man21	Y21-$(OH)_1$	2	2457.2062	nd	_	2457.2145	3.4
Ara1Man22	Y22-$(OH)_1$	2	2559.2561	nd	_	2559.267	4.3

nd-not detected

[a] The ions identified in Fig. 9

Supplementary Table 4. Assignment of fragment ions from the MS/MS analysis of the per-*O*-methylated Ara_2-Man_{24}-Inos-P-Gro at *m/z* 1906.27 $[M+(NH_4)_3]^{3+}$ (see Fig. 9).
